# Spring-mediated distraction enterogenesis may alter the course of adaptation in porcine short bowel syndrome

**DOI:** 10.3389/fgstr.2024.1292226

**Published:** 2024-03-18

**Authors:** Geoanna M. Bautista, Genia Dubrovsky, Nicolle K. Sweeney, R.S. Solórzano-Vargas, Daniel J. Tancredi, Michael Lewis, Mattias Stelzner, Martín G. Martín, James C.Y. Dunn

**Affiliations:** ^1^ Department of Pediatrics, Mattel Children’s Hospital and the David Geffen School of Medicine, University of California, Los Angeles, Los Angeles, CA, United States; ^2^ Department of Pediatrics, University of California Davis Children’s Hospital, Sacramento, CA, United States; ^3^ Department of Surgery, David Geffen School of Medicine, University of California, Los Angeles, Los Angeles, CA, United States; ^4^ Department of Pathology, Veteran's Affairs (VA) Greater Los Angeles Healthcare System, Los Angeles, CA, United States; ^5^ Department of Surgery, Veteran's Affairs (VA) Greater Los Angeles Healthcare System, Los Angeles, CA, United States; ^6^ Departments of Surgery and Bioengineering, Division of Pediatric Surgery, Stanford University School of Medicine, Stanford, CA, United States

**Keywords:** spring-mediated enterogenesis, short bowel syndrome (SBS), neonatal short bowel syndrome, intestinal lengthening, intestinal failure

## Abstract

**Introduction:**

Severe forms of short bowel syndrome (SBS) resulting in chronic intestinal failure (IF) have limited therapeutic options, all of which are associated with significant morbidities. Spring-mediated distraction enterogenesis (SMDE) uses an intraluminal self-expanding spring to generate mechanical force to induce intestinal stretching and sustained axial growth, providing a promising novel approach for patients with SBS. Previous studies have established this method to be safe and effective in small and large animal models. However, SMDE has previously not been implemented in a large, clinically relevant animal model.

**Methods:**

Juvenile mini-Yucatan pigs with 75% of their small intestine resected had intraluminal springs placed after an initial adaptive period. Morphological and histological assessments were performed on SMDE segments compared to the control region of the intestine undergoing normal adaptive responses to resection.

**Results:**

While the initial histologic adaptive response observed following resection was attenuated after a month, the SMDE segments instead augmented these adaptive changes. Specifically, intestinal length increased 2-fold in SMDE segments, and the widths of the epithelial, muscularis, and serosal layers were enhanced in SMDE compared with control segments of the same animal. This data suggests that morphologic intestinal adaptation may be enhanced with SMDE in the setting of SBS.

**Discussion:**

Here we demonstrate the successful and reproducible implementation of SMDE in a large animal model in the setting of prior intestinal resection, making SMDE a viable and novel approach for SBS to be explored further.

## Graphical Abstract

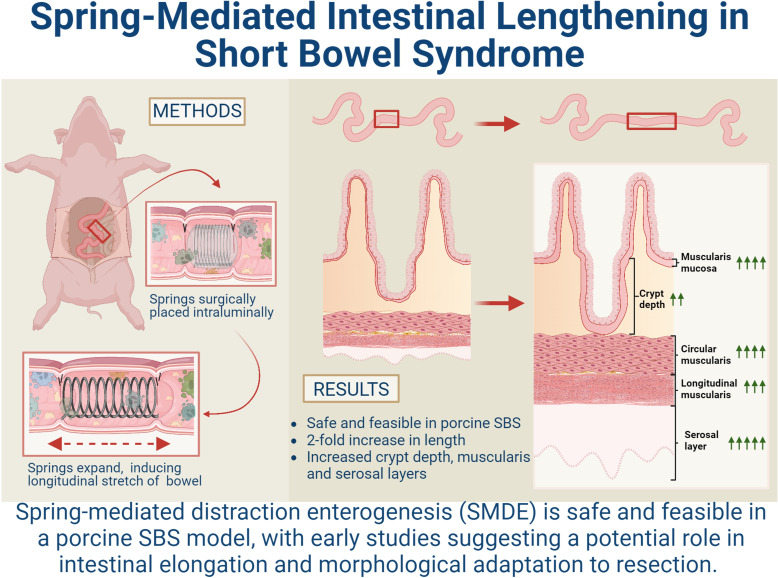

## Introduction

The leading cause of intestinal failure (IF) in neonates and older children is short bowel syndrome (SBS), typically following surgical resection due to various neonatal pathologies, including necrotizing enterocolitis, intestinal atresia, gastroschisis, and midgut volvulus (1, 2). While most neonates undergo intestinal adaptation and eventually achieve enteral autonomy, those with severe SBS have limited therapeutic options (3). These include life-long parenteral nutrition, surgical lengthening procedures, and bowel transplantation, all of which are associated with significant morbidity and mortality (3).

Teduglutide, an analog of glucagon-like peptide 2 (GLP-2), has more recently been used to promote intestinal adaptation in patients with persistent intestinal failure and is currently approved for use in children > 1 year of age (4). However, there are limited studies on children, with no long-term studies available (5). While teduglutide enhances intestinal adaptation, more data is needed to identify which patients would benefit most from treatment. In addition, current studies indicate that long-term administration is necessary to retain the enteral autonomy achieved, limiting its efficacy (6). Other trophic agents, such as glutamine and growth hormone, may enhance intestinal adaptation; however, none have yet been clinically efficacious for a prolonged duration (7).

Currently, available surgical lengthening procedures include longitudinal intestinal lengthening and tailoring (LILT), serial transverse enteroplasty (STEP), and spiral intestinal lengthening and tailoring (SILT) procedures (8, 9). However, while these methods have been shown to reduce the time to full enteral autonomy in some instances, there remains a high failure rate, particularly in the neonatal population, with associated morbidities including obstruction, perforation, bleeding, abscesses, and fistula development (8, 10). Thus, there continues to be a need to establish novel methods and interventions for severe SBS, potentially utilizing an integrated medical and surgical approach to achieve the best outcomes.

Distraction enterogenesis is a novel approach to bowel lengthening that uses mechanical force to stretch the intestine longitudinally to induce subsequent growth (11). Specifically, spring-mediated distraction enterogenesis (SMDE), in which an intraluminal spring generates the necessary force to induce stretch and intestinal growth, is a promising technique that is safe and effective in small and large animal studies. Previous studies using this method have demonstrated successful intestinal lengthening in rats (12–17), mice (18, 19), and pigs (20–23). Furthermore, SMDE has been shown to maintain normal gut functions, including absorption and peristalsis, in elongated segments of the intestine (24). The safety and feasibility of SMDE in a large SBS animal model have yet to be established. Therefore, we hypothesized that SMDE would be safe and feasible in a porcine model of SBS and that SMDE would sustain or enhance the expected intestinal adaptation following significant bowel resection. This study is the first reported attempt to lengthen the bowel in the setting of prior intestinal resection in a large animal model.

## Methods

### Animals

All experiments involving animals in this study were approved by the Animal Research Committee (# 2014-142-11). Female juvenile mini-Yucatan pigs weighing 8-10 kg and 5-6 weeks old were purchased from S&S Farms (Ramona, CA). All materials used in this study are FDA-approved for use in humans. Five animals were used for this feasibility study, three of which were shown in the final analyses included. The initial assessments on the first two pigs examined the characteristics of the springs, making necessary adjustments that were then implemented on the last three pigs. The primary modifications addressed the force generated by the spring to accommodate for the intestinal adaptation that occurred following resection, which was not present in the earlier, non-resected model, with technical adjustments made as described below. The remaining three animals underwent SBS surgery and SMDE without additional complications.

### Spring development

Springs were made from 0.02-gauge nickel-titanium (nitinol) wire (McMaster-Carr, Santa Fe Springs, CA). Springs were made over a 2.0 cm diameter mandrel with grooves. Nitinol wire with a 0.0315-inch diameter was wrapped around the mandrel for 5.5 active turns and heated to 500°C for 20 min, followed by rapid cooling via submersion underwater to generate an applied force of 20-22 N/m. These were placed inside size 13 gelatin capsules (Fisher Scientific, Pittsburg, PA). Capsules were then coated with dissolvable cellulose acetate phthalate (Eastman Chemicals, Fairfield, NJ). The initial spring characteristics were created to generate a force of 6.4 N/m and 14.5 N/m with an outer diameter of 1.2 cm, which did not adequately address the typical adaptive properties of the intestine following resection. Thus, the final springs used for analysis generated 20-22 N/m of force with an outer diameter of 2.0 cm, which was well tolerated in the three pigs used in the study.

### Surgery

**Figure 1:**
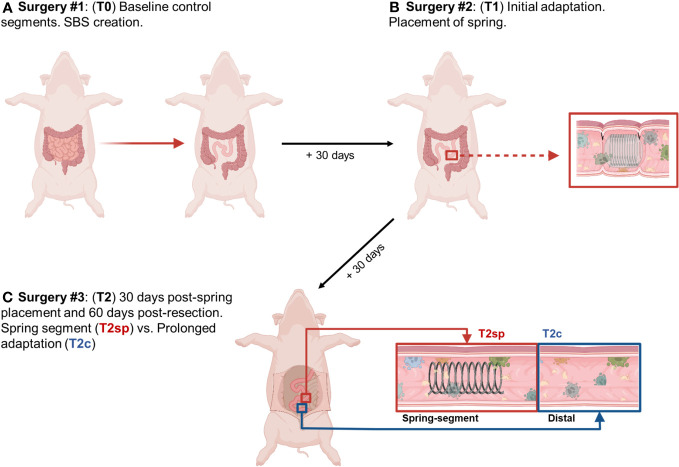
Illustrated schematic of experimental design. **(A)** 75% of small bowel resected, and baseline intestinal segments were obtained and labeled T0. **(B)** 30 days following resection, segments were obtained for analysis of initial adaptation and labeled T1. **(C)** Animals were sacrificed 30 days following spring placement and 60 days following initial bowel resection. Segments were obtained from the spring-induced stretched intestine (T2sp), and segments distal to spring as control and analyzed as prolonged adaptation (T2c). *Figure created with Biorender.com by G.M. Bautista*.

Pigs were anesthetized with inhaled isoflurane and intubated. Under sterile conditions, a midline incision was made, and the ligament of Treitz was identified. Approximately 75% of the small intestine (~550-650cm) was removed with a jejunoileostomy to create the SBS model (T0), consistent with previously established models (25) (**Figure 1**). 30-days following resection (T1), pigs underwent a second laparotomy to place an encapsulated compressed spring in the jejunal segment via plication with sutures as previously described (26). At T2 (30 days following spring placement and 60 days post-resection), pigs were sacrificed, and tissue was obtained from the site of spring placement and tissue proximal to the spring to serve as an internal control. Immediately postoperatively, the pigs were given liquid diets using Ensure formula to provide caloric needs while minimizing obstruction risk adequately. After passing normal stools, the pigs resumed their regular diets the day after intestinal surgery, growing appropriately without concern for obstruction.

### Histological analysis

Segments were prepared for histological evaluation, placed in 10% buffered formalin (Fisher Scientific, Pittsburg, PA) overnight, and subsequently embedded in paraffin. Slides were stained with human anti-desmin (muscle marker), Cdh1 (E-cadherin), Cd10 (Neprilysin), and Tuj (neuronal marker, anti-beta III tubulin)-antibodies and processed by the UCLA Pathology Department. Muscle layers were differentiated by striation patterns seen with desmin staining to capture the external muscularis layers (circular and longitudinal) and the muscularis mucosal layer, which underlies the epithelium. The thickness of each layer was subsequently measured and quantified.

The total epithelial length was measured using the Cdh1 stain. Since Cd10 specifically stains the villus, crypt depth was measured by the full epithelial length minus the villus length (Cdh1 – Cd10). Thus, the Cd10/(Cdh1 - Cd10) ratio was used to determine the relative portion of villus height and crypt depth contributing to the overall epithelial length. Histological measurements and analyses were performed using ObjectiveView. Measurements were taken every 10mm along the circumference of each bowel section, obtaining at least 20 representative areas from each slide.

### RNA extraction and RT-qPCR analysis

RNA was isolated from tissue using the Invitrogen PureLink RNA Mini Kit (cat #12183018A). RT-qPCR was conducted using Thermo Fisher 1-Step TaqMan Master Mix (cat #4392938) and TaqMan Probes for the following genes chromogranin A (ChgA), intestinal trefoil factor (Itf), lysozyme (Lyz), leucine-rich repeat-containing G-protein coupled receptor (Lgr5), and (glyceraldehyde-3-phosphate dehydrogenase (Gapdh). Data were analyzed using the ΔΔCT method relative to Gapdh expression using duplicates and averaged for final analysis.

### Statistical analysis

Statistical analysis was performed using GraphPad Prism (version 10.0.0). Measurements were log-transformed to improve the validity of the statistical inferences. Our randomized block design, with animals as blocks, permits the use of internal controls to account for animal-to-animal variation and a reduced number of animals needed to analyze between-group contrasts, which were estimated in two-way ANOVA models with animals as a blocking factor and condition as a within-animal factor. These contrasts were then transformed by applying the inverse log function, resulting in geometric mean ratios. Multiple comparisons were adjusted using Tukey’s *post-hoc* test. Data are presented as mean ± SEM or geometric mean ratio (GMR) with 95% CI unless otherwise indicated.

## Results

### Optimization of luminal spring

Springs initially used in this model were based on previously optimized methods used in healthy porcine models (22, 23, 26). However, following massive intestinal resection, the small intestine was more dilated and thickened than the historical controls. This required several modifications of the spring characteristics to generate optimal force without causing perforation or obstruction. Two initial pigs were used to assess the necessary adjustments in the SBS version of the porcine model (data not shown).

The characteristics of the final spring were adjusted to maintain the force per unit cross-sectional area as follows: the spring diameter was increased to 20 mm, the thickness of the wire gauge was increased to 0.80 mm wire gauge, and the coils underwent 5.5 active turns to generate a force constant of 20 N/m (increased from 6-8 N/m and 14 N/m in earlier assessments).

### Bowel resection initially leads to adaptive morphological changes in all layers

**Figure 2:**
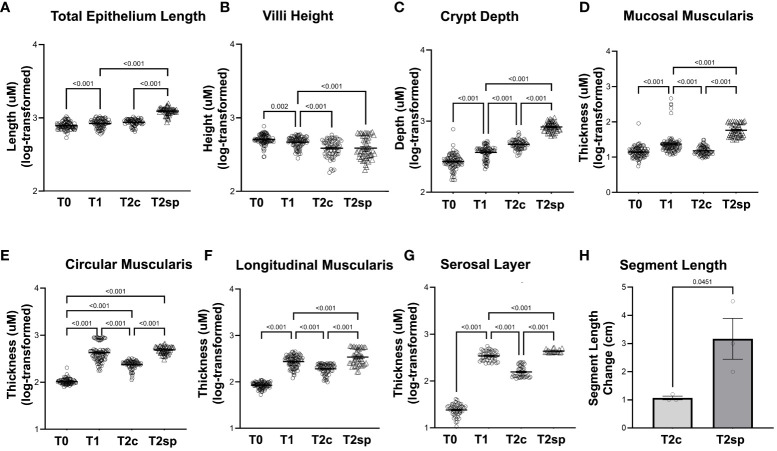
Morphological changes in adaptation and SMDE. SMDE leads to increased **(A)** total epithelium length, **(C)** crypt depth, **(D)** mucosal muscularis, **(E)** circularis muscularis, **(F)** longitudinal muscularis, and **(G)** serosal layer, but similarly decreased **(B)** villi height. **(H)** Total intestinal length is increased in SMDE segments (T2sp) 3-fold compared to control/prolonged adaptation (T2c). N=3 animal, n=30 regions obtained per time point. Data shown as mean+/-SEM of log-transformed data with p-values indicated above compared groups using Two-Way ANOVA analysis with Tukey's post-hoc analysis.

**Figure 3:**
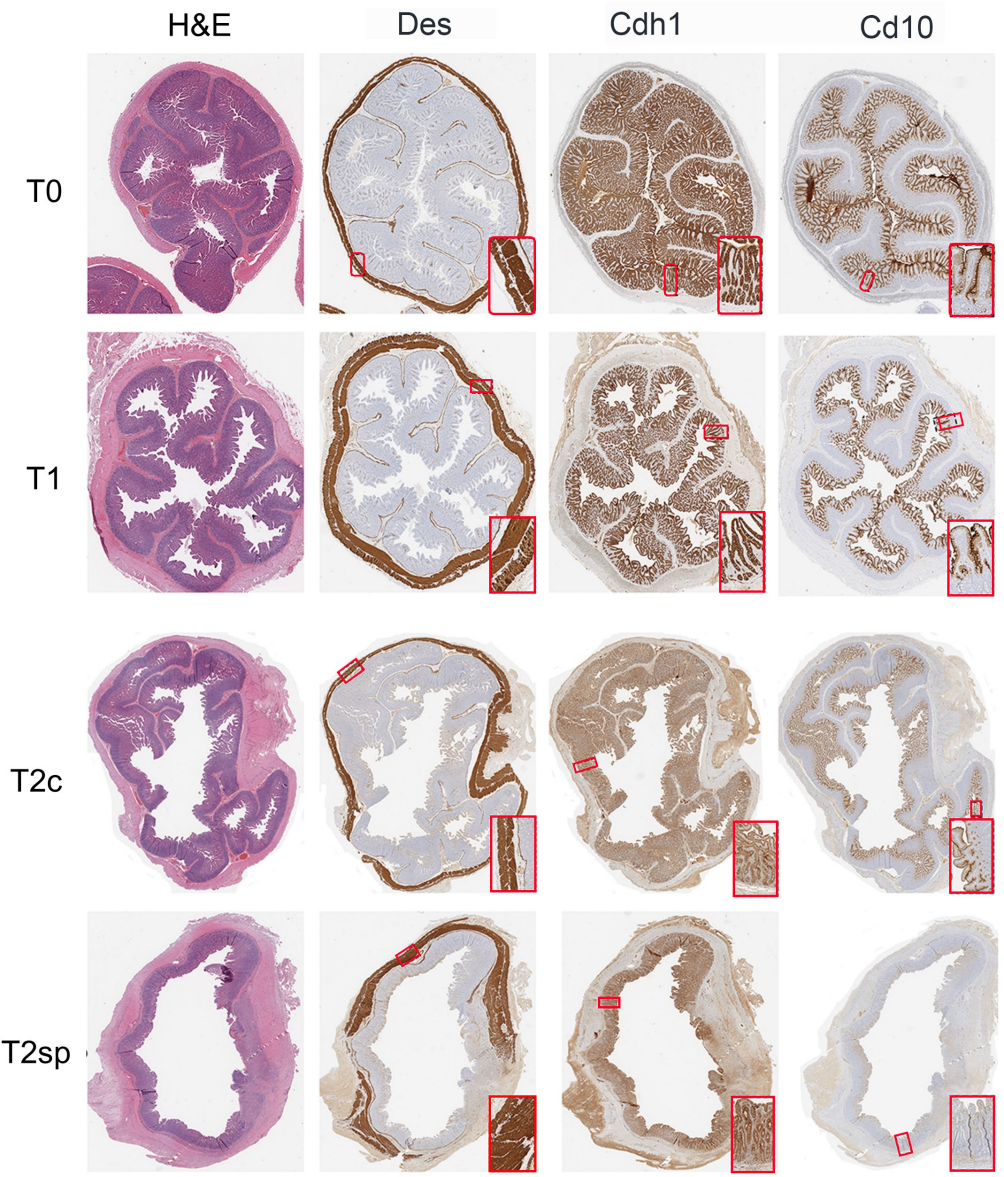
Representative histological sections. Intestinal samples obtained from each time point were stained for H&E, Des (muscle), Cdh1 (epithelium), and Cd10 (villi-specific epithelium).

To assess the adaptive responses of the intestinal layers following resection, histological assessments were made of the relative thickness of each layer at 30 days post-resection (T1) compared with baseline (T0) (**Figures 1**–**3**).

Following bowel resection during initial adaption at T1 compared to baseline at T0, total epithelial length (measured by Cdh1 staining) increased by 6% (GMR 1.06, 95%CI: 1.02,1.10 p<0.001), while the crypt depth (Cdh1-Cd10) significantly increased by 37% (GMR 1.37, 95%CI: 1.28,1.48 p<0.001). In contrast, villus height (Cd10) decreased by 10% (GMR 0.90, 95%CI: 0.83,0.97 p=0.002) (**Figures 2A–C**).

Changes in muscularis thickness were measured using desmin-positive staining. At T1 compared to T0, the geometric mean mucosal muscularis increased by 50% (GMR 1.50, 95%CI: 1.35,1.66, p<0.001), the circular muscularis increased by 337% (GMR 4.37, 95%CI: 3.95,4.82 p<0.001), and the longitudinal muscularis increased by 222% (GMR 3.22, 95%CI: 2.93, 3.54 p<0.001) (**Figures 2D–F**). The surrounding serosal layer was more dramatically increased by 14-fold (GMR 14.16, 95%CI: 12.01,15.52, p<0.001) at T1 (**Figure 2G**).

These data imply that increases in epithelial length following resection are driven primarily by crypt elongation rather than villus lengthening. Furthermore, the muscularis and serosal layers significantly increase following resection during the initial adaptation period.

### Intraluminal springs induce intestinal lengthening in a porcine SBS model

Springs were then surgically placed intraluminally 30 days post-resection (T1), and the bowel was assessed 30 days after placement (T2sp). Following surgical placement of the springs, the animal consumed standard porcine chow without any diarrhea or other signs of malabsorption, continuing to gain weight appropriately. The entire bowel length was examined at the time of tissue retrieval, and no obstruction was observed.

To account for the normal adaptation in intestinal resection and animal-to-animal variation, internal within-animal controls were used to determine the length induced by SMDE relative to control regions (23). The effect of the capsule alone without the spring was previously investigated in other animal models. No lengthening of the intestinal segment could be attributed to the capsule alone (23). India ink was used to mark 1.5 cm within the SMDE segments (T2sp) and intestinal segments distal to springs (T2c) (**Figure 2H**). T2sp segments increased to an average length of 3.17 +/- 1.26 cm from a starting measurement of 1.5 cm, whereas T2c segments decreased to 1.07 +/- 0.12 cm from 1.5 cm. The spring-associated segments were 2-fold greater than the control segments within the same animal (p<0.045). Thus, SMDE induced a 2-fold lengthening compared with baseline but 3-fold compared to control segments (p<0.01) (**Figure 2H**). These data suggest that SMDE overcomes the shortening that naturally occurs following intestinal resection.

### Prolonged morphological adaptive response to bowel resection tapers off

Morphological and structural changes were assessed 60 days post-resection of control segments (T2c). The initial growth observed within the muscularis and serosal layers was reversed during T2c, with the mucosal muscularis layer decreasing by 31% (GMR 0.69, 95%CI: 0.62, 0.78 p<0.001), the circular layer decreasing by 50% (GMR 0.50, 95%CI: 0.45, 0.56 p<0.001), the longitudinal muscularis decreasing by 33% (GMR 0.67, 95%CI: 0.60, 0.74 p<0.001), and the serosal layer decreasing by 51% (GMR 0.49, 95%CI: 0.44, 0.54 p<0.001) relative to T1 (**Figures 2**, **3**).

Similarly, crypt depth continued to increase by 27% at T2c (GMR 1.27, 95%CI: 1.17,1.37 p<0.001) relative to T1 (**Figure 2C**). Villus length continued to decline by 18% at T2c (GMR 0.82, 95%CI: 0.75, 0.89 p<0.001) (**Figure 2B**). These data suggest that after a prolonged period following resection, the epithelial layers continue to undergo adaptation, albeit at an attenuated rate. In contrast, the muscularis and serosal layers reverse the initial adaptive processes by 60 days post-resection.

### SMDE enhances the morphological adaptive responses to bowel resection

Compared to the control, non-stretched jejunal segments proximal to spring placement (T2c), and SMDE segments (T2sp), the mucosal muscularis increased by 288% compared to T2c (GMR 3.88, 95%CI: 3.43,4.40 p<0.001) and 169% higher relative to T1 (GMR 2.69, 95%CI 2.38,3.04 p<0.001). The circular layer increased by 105% compared to T2c (GMR 2.05, 95%CI: 1.83, 2.30 p<0.001). The longitudinal layer was increased by 78% compared to T2c (GMR 1.78, 95%CI: 1.57, 2.01 p<0.001) and by 19% from T2 (GMR 1.19, 95%CI: 1.05, 1.34 p<0.001). The serosal layer was significantly increased by 205% compared to T2c (GMR 3.05, 95%CI: 2.68, 3.48 p<0.001) and by 49% from T2 (GMR 1.49, 95%CI: 1.31,1.69 p<0.001).

Similarly, measurements within the non-stretched jejunal segments proximal to spring placement and SMDE segments were significantly increased across all the intestinal layers except the villi (**Figures 2**, **3**). Within the epithelium, compared to T2c, SMDE led to significantly increased crypt depth by 75% (GMR 1.75, 95%CI: 1.60, 1.91 p<0.001), with an overall 121% increase (GMR 2.21, 95%CI: 2.03, 2.41 p<0.001) relative to T1 (**Figure 2C**). These data suggest that SMDE enhances morphological responses to resection and can alter the eventual morphologic attenuation that occurs with prolonged adaptation.

### Cell populations during adaptation following resection are altered with SMDE

**Figure 4:**
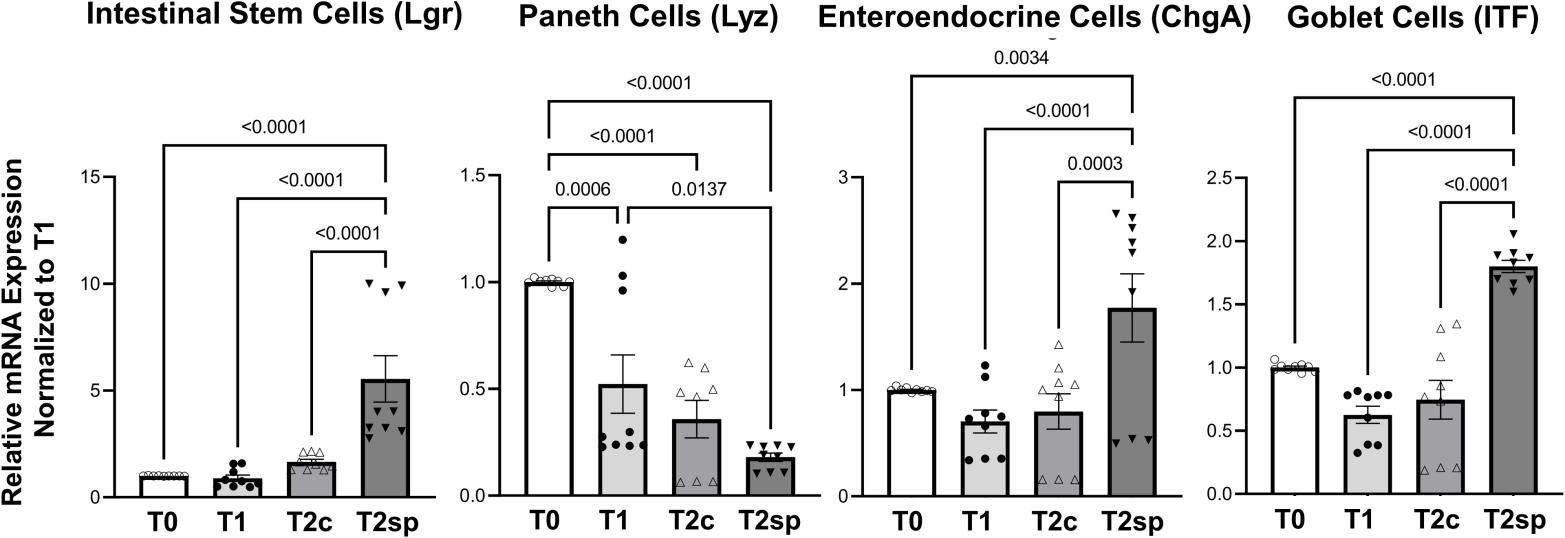
Relative mRNA expression levels of specific cell populations. SMDE (T2sp) significantly increases the expression of Lgr5+ (stem cells), Chga (enteroendocrine cells), and Itf (Goblet cells) and decreases the expression of Lyz (Paneth cells). N = 3 animals, n = 3 biological replicates per animal per time point. Data are shown as mean+/-SEM with p-values indicated above compared groups using Two-Way ANOVA analysis with Tukey’s posthoc analysis of mRNA levels relative to baseline using housekeeping gene of gapdh.

To further characterize the increase in crypt expansion, mRNA levels of Lgr5, a marker for intestinal stem cells (ISC), were measured across the same animal’s different time points. ISCs were significantly increased in the spring segments when compared to baseline adaptation at T2sp by 4.95 (95%CI: 2.68, 6.61 p<0.0001) and when compared to control segments (T2c) within the same animal by about 3.88 (95%CI: 1.92, 5.85 p<0.0001) (**Figure 4**). In contrast, Lyz, a marker for Paneth cells which also reside within the crypts with the ISCs, were significantly decreased with a mean reduction of 0.48 95%CI: 0.76, 0.19 p=0.0006) at T2c compared to baseline at T1. There was a further decline in Lyz mRNA expression in both controls (T2c) (0.65, 95%CI:0.95, 0.36, p<0.0001) and SMDE (T2sp) segments (0.82 95%CI: 1.10, 0.537, p<0.00001) compared to T1.

In contrast, ChgA (chromogranin), a marker for enteroendocrine cells, and ITF, a marker for mucin-producing Goblet cells, were significantly enhanced in SMDE segments at T2sp. Specifically, ChgA in SMDE segments was increased by 1.069 (95%CI: 0.524, 1.614, p<0.0001) compared to T2c and by 0.975 (95%CI: 0.430, 1.520, p=0.0003) compared to control segments (T2c). ITF expression increased by a mean of 1.174 (95%CI: 0.796, 1.552, p<0.0001) compared to T1, with a similar increase of 1.056 (95%CI: 0.677, 1.433, p<0.0001) (**Figure 4**). These data suggest that at this specific time during adaptation, the intraluminal springs result in increased goblet cell and enteroendocrine, which are essential for normal gut function. The decreased expression of Paneth cells throughout normal adaptation and with SMDE suggests that these cell types may be produced at a later stage, similar to the expected timing of gut maturation. Lastly, staining with Tuj1 was performed to illustrate the continuation of enteric neuronal activity in the setting of SBS and SMDE, but was difficult to quantify (**Supplementary Figure 1**).

## Discussion

This study aimed to explore the safety and feasibility of SMDE in a clinically relevant SBS porcine model, highlighting morphological changes unique to this setting. Following extensive small bowel resection, structural and functional changes occur to compensate for the sudden loss of absorptive and digestive capacity in the remaining intestine. Interestingly, this study found that despite an overall increase in crypt depth and total epithelial length, the actual villus height, when stained more specifically, decreased 30 and 60 days following resection. Despite this apparent villus shortening, the pigs did not display any signs of malabsorption or diarrhea. They continued to gain appropriate weight, suggesting that the villi may have been captured when they had not yet reached their total growth, as evidenced by the ongoing crypt elongation. This highlights the dynamic adaptation process and that the historically characterized structural changes may occur at different phases.

These data also show that despite early and persistent crypt expansion, Lgr5 mRNA expression did not significantly increase until 60 days following resection in the stretched SMDE segments. This is consistent with recent data highlighting the transient peaks of intestinal growth and the activation of different signaling pathways driving stem cell expansion and new crypt formation that will subsequently cause epithelial regeneration and repopulation, including villus growth (27). These results may also suggest that expansion of other cellular components of the crypt, such as transient amplifying (TA) or early progenitor cells, or that the presumed expansion of the ISC population was not Lgr5 but perhaps Lgr4 or Lgr6. Unfortunately, Lgr5 and Olfm4 immunostaining was unsuccessful on porcine small bowel samples. This significant expansion of the crypts relative to the villi likely suggests a relative depletion of differentiated cell populations, particularly enterocytes.

Interestingly, the morphological changes noted in the early adaptation phase began to taper off 60 days following resection. Specifically, the external muscularis layers started to lose some of their initial growth, and the muscularis mucosal layer returned to baseline height by T2c. This appears to have been altered with SMDE (T2sp), which continued to drastically increase the growth of all three muscularis layers beyond the initial adaptive changes. This suggests that SMDE may help maintain the intestinal rehabilitation necessary for prolonged enteral adaptation and autonomy, potentially mitigating the eventual attenuation that would have occurred otherwise, particularly within the muscularis layers. The dramatic growths of the muscularis mucosa and the serosal layers, coupled with the significant increases in the crypt and stem cell expansion in SMDE segments, are particularly notable given that recent studies have illustrated the emerging importance of the muscularis layers in driving signaling processes essential for normal growth and maturation of the epithelial layers (28, 29). These findings suggest that the durable increase in intestinal length may enhance the morphological changes occurring with adaptation. Critical mechanotransduction signaling pathways may be triggered by the longitudinal stretch induced by the intraluminal springs. Additional studies are still necessary to further characterize the signaling mechanisms driving these adaptive processes and how SMDE can potentially augment these changes to reach enteral autonomy in severe SBS.

While this study highlighted the safety, feasibility, and morphological impact of SMDE, we could not provide meaningful functional assessments due to the limited sample size and lack of available reagents at the time. Thus, several of our assessments were limited to mRNA expression data due to the limited availability of validated antibodies in the porcine model. Therefore, a more comprehensive evaluation with a greater sample size will be necessary to delineate the true impact of SMDE in SBS fully. Future studies are underway to assess further the re-establishment of functional and absorptive capacities of the elongated intestine, and long-term studies are being conducted to determine the reversibility of the achieved length after the springs are removed.

Despite these limitations, the findings of this study have implications regarding the use of SMDE to enhance the ongoing adaptive processes in bowel resection. Furthermore, our data suggest that there may be a benefit for the earlier implementation of distraction enterogenesis, particularly with the development of less invasive insertion methods, to aid in the adaptive processes of residual bowel.

In conclusion, spring-mediated distraction enterogenesis is feasible and safe in a large animal SBS model, inducing functional intestinal growth while maintaining luminal patency. Additionally, this study highlights the importance of mechanical stretch in enhancing the normal adaptive processes already occurring in the setting of SBS, providing a foundation for further development with currently available therapeutic interventions for severe SBS and IF. Additional studies are required to fully elucidate the mechanisms that drive the changes induced by SMDE, with attention to long-term studies of safety and continued enterogenesis.

## Data availability statement

The raw data supporting the conclusions of this article will be made available by the authors, without undue reservation.

## Ethics statement

The animal study was approved by Animal Research Committee # 2014-142-11. The study was conducted in accordance with the local legislation and institutional requirements.

## Author contributions

GB: Data curation, Formal analysis, Methodology, Resources, Validation, Visualization, Writing – original draft, Writing – review & editing. GD: Data curation, Investigation, Methodology, Writing – review & editing. NS: Formal analysis, Validation, Writing – review & editing. RS: Data curation, Investigation, Writing – review & editing. DT: Formal analysis, Validation, Writing – review & editing. ML: Data curation, Supervision, Writing – review & editing. MS: Data curation, Investigation, Methodology, Supervision, Writing – review & editing. MM: Formal analysis, Funding acquisition, Investigation, Methodology, Resources, Supervision, Validation, Visualization, Writing – review & editing. JD: Conceptualization, Data curation, Formal analysis, Funding acquisition, Investigation, Methodology, Resources, Supervision, Validation, Visualization, Writing – review & editing.

## Supplementary material

Supplementary Figure 1 Enteric neuronal staining. Intestinal samples obtained at each time point was stained with anti-tubulin III (Tuj1), showing continued expression of enteric neuron activity in the setting of SBS and SMDE.
